# Early Development of the Gut Microbiota and Immune Health

**DOI:** 10.3390/pathogens3030769

**Published:** 2014-09-24

**Authors:** M. Pilar Francino

**Affiliations:** 1 Unitat Mixta d’Investigació en Genòmica i Salut, Fundación para el Fomento de la Investigación Sanitaria y Biomédica de la Comunitat Valenciana (FISABIO)-Salud Pública/Institut Cavanilles de Biodiversitat i Biologia Evolutiva (Universitat de València), València 46020, Spain; E-Mail: francino_pil@gva.es; Tel.: +34-961-925-953; 2 School of Natural Sciences, University of California Merced, CA 95343, USA

**Keywords:** human microbiome, gut microbiota, immune disease, atopy, necrotizing enterocolitis, infant gut, early programming, microbe-host interactions, intrauterine transmission, antibiotics

## Abstract

In recent years, the increase in human microbiome research brought about by the rapidly evolving “omic” technologies has established that the balance among the microbial groups present in the human gut, and their multipronged interactions with the host, are crucial for health. On the other hand, epidemiological and experimental support has also grown for the ‘early programming hypothesis’, according to which factors that act *in utero* and early in life program the risks for adverse health outcomes later on. The microbiota of the gut develops during infancy, in close interaction with immune development, and with extensive variability across individuals. It follows that the specific process of gut colonization and the microbe-host interactions established in an individual during this period have the potential to represent main determinants of life-long propensity to immune disease. Although much remains to be learnt on the progression of events by which the gut microbiota becomes established and initiates its intimate relationships with the host, and on the long-term repercussions of this process, recent works have advanced significatively in this direction.

## 1. Intrauterine Transmission of Maternal Bacteria

One of the recent surprises regarding gut colonization is the fact that this process may be starting *in utero*. The *in utero* environment has been traditionally considered sterile under normal conditions, with microbial colonization of the gut beginning at birth, when the neonate is exposed to an avalanche of microbes, starting with those of the maternal vaginal and fecal microbiotas. However, culture-dependent analyses have detected microorganisms in amniotic fluid [1,2,3,4,5,6,7], fetal membranes [3], umbilical cord [8] and placenta [9], even in cases where no rupture of membranes has occurred [4,6] and in elective C-sections [8]. Live bacteria have also been isolated from human meconium, the newborn’s first intestinal discharge, composed of material that has been ingested or secreted in the gut during fetal life [10,11]. Although it is difficult to completely rule out the possibility of external bacterial contamination of intrauterine human samples, experimental work in mice has demonstrated an efflux of bacteria from the mother’s gut to that of the fetus. To this aim, Jiménez *et al.* [10] orally inoculated pregnant mice with a genetically labeled *Enterococcus faecium* strain, and were then able to isolate and PCR-detect this strain from the meconium present in the offspring, obtained by C-section one day before the predicted date of labor; in contrast, the strain could not be detected in samples obtained identically from a non-inoculated control group. Such bacterial efflux could be facilitated by the enhanced bacterial translocation from the gut to mesenteric lymph nodes that takes place during late pregnancy [12], followed by bacterial entrance into the systemic circulation. The fact that peripheral blood mononuclear cells of lactating women have been shown to carry a wide range of bacterial DNA signatures, and, in a fraction of cells, intact bacterial bodies [12], suggests that transport of bacteria in blood and lymph could occur within some subset of these immune cells.

More recently, several works have applied high throughput 16S rRNA gene pyrosequencing to investigate the diversity of the microbiota present in meconium [11,13,14,15,16,17]. These analyses have revealed the presence at this stage of substantially diverse assemblages of bacteria. In most cases, the taxonomic composition of the meconium microbiota has not been found to depend on the neonates’ mode of delivery [14,16,17], with exception of the study by Domínguez-Bello *et al.* [13], in which samples were obtained by means of rectal swabs after meconium passage, rather than by the collection of the entire first intestinal discharge of the newborns. In this case, the composition of rectal swab samples was undistinguishable from that of samples from other areas of the neonates’ bodies, such as skin, oral mucosa and nasopharyngeal aspirate, and closely resembled vaginal or skin microbiota, depending on the mode of delivery. This is in sharp contrast to the results of Gosalbes *et al.* [16], where the taxonomic composition of the meconium microbiota, both from vaginally and C-section-delivered babies, clearly differed from that of the microbiotas that could most easily contaminate the meconium during or shortly after childbirth, such as those present in the vagina, feces or skin of pregnant women. Rather, this study detected similarities in composition between the microbiota in meconium and that present in the infant during the first weeks of life, and some meconium strains were shown to remain in the infant gut up to seven months of age. These findings support the notion that the meconium microbiota has an intrauterine origin and participates in gut colonization.

The proposition that microbes pass in a controlled manner into the circulation of healthy individuals is groundbreaking but potentially controversial, as it challenges the notion that translocation of bacteria should lead to sepsis. Moreover, the implications of *in utero* microbial colonization for human health are likely to be enormous, as the bacteria reaching the fetal gut could strongly mark the trajectories of immune, metabolic and somatic development. Therefore, it will be paramount in upcoming years to understand the extent to which *in utero* transmission of maternal microbes occurs, which maternal factors influence this transmission process and the impact that it has on human development and health.

## 2. Compositional and Functional Development of the Gut Microbiota during Infancy

From birth, newborns will encounter a panoply of different microorganisms, some of which will be incorporated into their gut microbiotas. Use of 16S rRNA pyrosequencing has revealed that the microbiotas from newborn babies shortly after birth are quite similar across most body habitats; in vaginally-delivered infants, they resemble their own mother’s vaginal microbiota, dominated by *Lactobacillus*, *Prevotella*, or *Sneathia*, whereas in infants born by C-section they are most similar to the microbiota found on skin, dominated by *Staphylococcus*, *Corynebacterium*, and *Propionibacterium* [13]. However, within the first week of life, the gut develops a very uneven bacterial community, often heavily dominated by one or a few taxa, usually belonging to the genera *Escherichia*, *Clostridium*, *Bacteroides* or *Bifidobacterium* [18,19]. Throughout the first year of life, the gut microbiota changes in composition and increases in diversity, converging slowly towards the microbiota of the adult. Among breast-fed infants, *Bifidobacterium*-dominated microbiotas are more frequent than among infants fed with formula, but other compositions are also common. A large shift in microbiota composition accompanies the introduction of solid foods into the diet, although the specific taxa present before and after this point in different infants will vary [18,19,20]. By one year of age, the microbiota of infants substantially resembles that of the adult, being dominated by the phyla Bacteroidetes and Firmicutes, mainly represented by the genera *Bacteroides, Faecalibacterium, Clostridium* and *Ruminococcus* [19]. Nevertheless, a typically adult pattern is probably not established until children reach the age of three [21].

Functional characterization of the infant gut microbiome lags behind studies of phylogenetic composition, but recent metagenomic analyses have initiated this task, showing that, in spite of taxonomical disparities, the infant rapidly acquires a functional gene repertoire dominated by carbohydrate metabolism genes and broadly similar to that of the adult. Nevertheless, the functional repertoire of the infant changes through the first year of life, as the earliest microbiota is enriched in genes facilitating lactate utilization, whereas solid foods promote enrichment in genes coding for the utilization of a larger variety of carbohydrates, vitamin biosynthesis and xenobiotic degradation [18,19,21,22,23,24].

## 3. Associations of Early Gut Microbiota with Immune Diseases

Undoubtedly, one of the main routes through which the early microbial colonization of the gut will shape life-long health is its close and determinant interaction with the immune system. In fact, achieving an adequate balance in the process of gut colonization may be the prime requirement for proper immune modulation and induction of immunological tolerance, the failure of which will result in the onset of autoimmune or atopic diseases [25,26,27]. The prevalence of these disorders in industrialized societies has greatly augmented in the last decades. Epidemiological data indicate that this rise may be linked to environmental and lifestyle changes that have reduced our exposure to microbes during infancy [28]. Indeed, infants who are more exposed to microbes, by having higher numbers of siblings or household pets, attending group day care at an earlier age or being raised in farms, have a much lower incidence of atopic disease [28,29,30,31]. These associations suggest that a reduced exposure to microbes results in altered gut colonization patterns that increase the likelihood of immune imbalances and associated disorders [27].

### 3.1. Atopic Disease

Numerous studies have shown that the specific composition of the gut microbiota during infancy and early childhood is linked to the relative occurrence of different types of atopic disease [25,32,33,34,35,36,37,38,39,40]. A first association was noted in the early 1980s, when Kuvaeva *et al.* [41] reported a deficiency of bifidobacteria and lactobacilli combined with an increase of enteric bacteria in children suffering from food allergies in Russia. Since then, several studies in Northern Europe, Japan and Singapore have confirmed a statistically significant link between bifidobacteria deficiency and an increased incidence of atopy, on the basis of bacteriological culture [32,42,43], FISH [34,44] and 16S rDNA real-time PCR [38]. However, two large prospective case-control studies have failed to confirm such an association [35,45] and conflicting results have been obtained regarding the protective role of different *Bifidobacterium* species [38,46,47]. In the case of lactobacilli, and in spite of their demonstrated immunoregulatory properties *in vitro* [48,49,50,51,52,53], only studies carried out in Sweden and Estonia have been able to confirm their protective role regarding atopy [32]. These discrepancies may be due to the presence of different bacterial species and strains in different geographical regions, and to genetic differences among human populations. Regarding potentially harmful microbes, clostridia have been associated with atopic diseases in numerous studies [33,34,36,54,55,56]. In particular, the *Clostridium coccoides* group has been found to be overrepresented in the feces of infants suffering from cow-milk protein allergy [57]. On the other hand, high abundances of *E. coli* and other enterics have been repeatedly linked to eczema [36,37,39,40], as well as to other allergies [33,43,44]. In addition, altered fecal levels of bacterial fermentation products, such as short-chain fatty acids, including butyric and valeric acids, have also been associated with atopy, particularly in food allergies [57,58]; these findings indicate that the metabolic functionality of the gut microbiota is related to the development of atopy.

Furthermore, analyses of the microbiota present in meconium have indicated that the association between high abundances of enteric bacteria and eczema may extend back to the intrauterine stage and may be initiated by maternal factors [16]. Indeed, in a Spanish children cohort study, there was an increased abundance of enterics in the meconium of children who developed eczema by four years of age or whose mothers had a history of eczema. In addition, interestingly, enteric bacteria were also more abundant in children whose mothers smoked during pregnancy, which is known to affect immune system development and to increase the risk of eczema in the offspring. Moreover, bacterial diversity was lowest in the meconium microbiotas when the abundance of enterics was elevated, and low diversity in the intestinal microbiota of infants during the first weeks of life has also recently been strongly linked to the presence of eczema [37,40].

### 3.2. Necrotizing Enterocolitis

A variety of disorders related to inflammation and autoimmunity have also been linked to the composition of the gut microbiota. In infants, the most devastating inflammatory bowel disease (IBD) is necrotizing enterocolitis (NEC), the incidence of which increases with decreasing gestational age at birth. In preterm newborns, NEC affects 10% of infants under 28 weeks of age with a mortality rate of 50%, and it is also becoming implicated in subsequent neurodevelopmental defects, including cerebral palsy and microcephaly [59]. The gut microbiota likely contributes to NEC etiology, as reduced diversity and low *Bifidobacterium* counts have been observed prior to onset of the disease [60,61].

## 4. Cellular and Molecular Mechanisms of Gut Microbiota Interaction with the Immune System

The impact of alterations in gut microbiota development on immune health is not surprising, given the numerous ways in which bacteria interact with the immune system and modulate its activity. Innate immunity, which performs the earliest recognition of microbes, is most developed in the intestinal tract, where a variety of immune and epithelial cells encode receptor molecules for ligands of microbial origin, such as capsular polysaccharides (PSA) and lipopolysaccharides (LPS), peptidoglycan, muramic acid, flagellin and the unmethylated CpG motifs characteristic of bacterial DNA [38,62]. In response to such ligands, cytokines are produced that shape the differentiation of the naïve T cells of the adaptive immune system, which can differentiate into regulatory cells (Tregs) or into different types of helper cells (Th), mostly Th1, Th2 and Th17 [62,63]. Treg cells suppress the activation and development of naïve T cells towards Th types [63,64,65], and have a variety of anti-inflammatory roles, including suppression of the inflammatory activities of mast cells, basophils and eosinophils [66], supression of immunoglobulin (Ig) E and induction of IgG4 [67]. On the other hand, each type of Th cell plays a distinct and key role in further directing and amplifying the immune response [49,63,68], while producing a cytokine profile that can suppress other Th types [69,70].

For a long time, mutual regulation between Th1 and Th2 cells (the Th1/Th2 balance) was considered to be the most critical factor for immune homeostasis. Indeed, excessive Th1 or Th2 activation results in chronic inflammatory and autoimmune disease or in allergic disease, respectively [71,72]. However, new lines of evidence indicate major roles for Th17 cells, which seem to be important in diseases that had classically been defined as Th1 or Th2-mediated [71,73,74], and for Tregs and their anti-inflammatory actions [75]. In this view, an inadequate microbial colonization of the gut results mainly in an imbalance between Treg cells and their effector targets, the different Th cells, and the subsequent deregulation of immune responses can promote a variety of pathological outcomes [36,76,77,78,79].

Experimental work has demonstrated that crosstalk between the gut microbiota and the immune system is indeed obligatory for the generation of Tregs. Treg cells are rare among lymphocytes recovered from germ-free mice [80], and different bacteria -including *Lactobacillus*, *Bifidobacterium*, *Bacteroides, Clostridium* and *Streptococcus*- and bacterial products [81] have been shown to induce Treg cells in various mouse models or *in vitro* cell cultures [82]. In contrast, work in mice has shown that the Segmented Filamentous Bacteria (SFB) induce the development of the pro-inflammatory Th17 cells [83], underlining how different commensal microbes can induce the differentiation of naïve T cells into different subtypes with different roles. However, SFB are not commonly encountered in the human gut microbiota, and there is no evidence that these bacteria play any relevant role in humans. In addition, the gut microbiota is also likely to influence immunotolerance development through its effects on the production of noninflammatory IgA, which contributes to pathogen and allergen exclusion in the intestinal epithelia, mucus and lumen [53,77,84].

## 5. Effects of Antibiotic Use

Given the main roles of the gut microbiota in immune homeostasis, it follows that antibiotic administration bears the risk of altering this basic equilibrium by affecting microbiota composition. This consideration has caused a growing concern that widespread use of antibiotics, especially at an early age, may have unintended long-term health consequences. The use of peripartum antibiotics has become particularly prevalent in modern obstetric and neonatal practice [85]. Nevertheless, there have been relatively few studies aimed specifically at gauging the effect of early antibiotic exposure on the development of the gut microbiota. Tanaka *et al.* [86] studied the microbiota of infants treated with cefalexin, a broad-spectrum antibiotic, during the first four days of life, and noted immediate effects within the first week, as well as longer-term effects within the following two months. The infants’ microbiota had a reduced diversity as well as alterations in composition during the first week, including an attenuation of *Bifidobacterium* and an increase of *Enterococcus*, and a marked increase of enterobacteria up to the second month. Interestingly, untreated infants whose mothers did receive antibiotics prior to delivery sustained similar microbiota alterations to the treated infants, although relatively weaker. Similarly, Fouhy *et al.* [87] analyzed the consequences of an ampicillin and gentamycin treatment within the first two days of life, and detected compositional alterations one month after the cessation of treatment, mainly an increase of the phylum Proteobacteria and decreases of the genera *Lactobacillus* and *Bifidobacterium* and of the phylum Actinobacteria. By two months, Proteobacteria levels remained elevated, but the Actinobacteria, *Bifidobacterium*, and *Lactobacillus* levels had recovered, even if the richness of species had been reduced.

There is also increasing evidence pointing towards consequences of early antibiotic use on immune health. Notably, the incidence of NEC increases in populations exposed to antibiotics, such as preterm newborns [88] and infants whose mothers receive antepartum antibiotics for attempted prolongation of pregnancy [89]. Longer-term consequences are also likely. Crohn’s disease, an IBD associated to an excessive and continuous stimulation of the mucosal immune system by the gut microbiota, has been shown to increase in children treated with antibiotics during the first five years of life [90]. On the other hand, the association of early antibiotic use with increased risk of asthma and allergies has been controversial [85,91]. Retrospective epidemiological studies have generally supported the association [92,93,94,95,96,97,98], but most prospective analyses have failed to do so [99,100,101,102,103,104,105]. Often, these analyses detected associations with antibiotic use that faded away after accounting for potential confounders such as early life respiratory infections [103], medical visits [105] or allergic symptoms in the first year of life [104]. Nevertheless, several prospective studies applying diverse strategies to reduce biases and confounding effects [106,107], including the largest prospective study to date [108], have been able to detect a positive association between asthma and early life exposure to antibiotics. These three large studies investigated the dose-response relationship between antibiotics and asthma, and revealed that the risk of asthma development increased with the number of antibiotic courses taken during the first year of life. This dose-response relationship lends strong support to the association of asthma with early antibiotic use. McKeever *et al.* [106] and Kozyrskyj *et al.* [107] also demonstrated that the association with asthma was stronger for broad-spectrum antibiotics, supporting the notion that the effect of antibiotics on asthma development acts through its depletion of diversity in the symbiotic microbiota. More recently, the prospective study by Risnes *et al.* [109] has also detected associations between early antibiotics and other allergic outcomes in addition to asthma. Furthermore, antenatal antibiotic intake also affects the risk for atopic disease, as the prevalence of asthma, hay fever and eczema has been found to increase in a dose-dependent manner with the number of antibiotic courses given to the mother during pregnancy [110].

Animal studies have enabled a better characterization of the effects of early antibiotic exposure on the gut microbiota, as well as probing of the possible mechanisms through which they alter the risk for immune disease. Sub-therapeutic antibiotic levels in young mice result in substantial taxonomic changes in the gut microbiome, changes in copies of key genes involved in the metabolism of carbohydrates to short-chain fatty acids, increases in colonic short-chain fatty acid levels, and alterations in the regulation of hepatic metabolism of lipids and cholesterol [111]. In addition, experiments with weeks-old mice have clearly demonstrated that antibiotics can bias immune development towards an atopy-prone state, as they promote cytokine profiles that maintain a Th2 phenotype [112,113]. In addition, antibiotics can also elevate the inflammatory tone of the intestine. Metronidazole has been shown to reduce the intestinal expression of Muc2, the major component of mucin [114], and the ensuing thinning of the mucus layer will increase contact between epithelial cells and the microbiota, enhancing immune stimulation and inflammation.

## 6. Effects of Preterm Birth and Delivery Mode

The process of early gut colonization is extremely variable among individuals and is influenced by numerous factors [13,115,116,117]. Among these, the gestational age and mode of birth of an infant are known to be important determinants of microbiota development.

In premature infants, a suite of related factors will perturb the establishment of the microbiota, including the immaturity of the immune response, the lack or delay of enteral feeding, and the repeated and often prolonged exposure to broad-spectrum antibiotics [61]. As a result, the gut microbiota of preterm infants contains a lower diversity of taxa and a reduced proportion of strict anaerobes with respect to facultatives during the first three months of life. In particular, the presence of *Bifidobacterium* is highly reduced, in favor of facultative anaerobes, such as *Enterococcus*, *Streptococcus*, *Staphylococcus*, and diverse enterobacteria [118,119,120]. Furthermore, the diversity of the microbiota decreases with lower gestational age and with longer periods of antibiotic treatment and parenteral nutrition [119]. The altered pattern of microbiota development in preterm infants contributes to impair the integrity of the intestinal mucosal barrier, which results in increased microbial translocation and permeability to microbial components, and, consequently, in higher risks of life-threatening outcomes, such as NEC, systemic inflammatory response syndrome, sepsis, and multisystem organ failure [61,121,122].

Birth by C-section also alters the pattern of gut microbiota development relative to that of vaginally born infants. As mentioned above, the very first microbial inocula received by the newborn are very different in each case, as the infants born by C-section are not exposed to their mother’s vaginal and fecal microbiota, and become initially colonized by skin bacteria, probably acquired from the surrounding environment [13]. Although most vaginal and skin bacteria do not seem to take hold in the infant gut, their presence may differentially affect the colonization capacities of other bacteria. Indeed, in surveys performed at three days and one month of age, infants born by C-section were much less likely to be colonized by *Bifidobacterium* and *Bacteroides* at both times, whereas the presence of *C. difficile* at one month was enhanced [117,123]. Underrepresentation of *Bacteroides* has also been detected in the microbiota of C-section infants surveyed at three-four months of age, along with lower numbers of *Escherichia*-*Shigella*. Interestingly, the same study detected that infants born by elective C-section had particularly low bacterial richness and diversity [124]. Longer-term studies have demonstrated that differences between the microbiotas of C-section and vaginally born infants can still be detected by seven years of age [125]. In line with the crucial role of early microbial inputs for infant immune development and health, various cytokines implicated in neonatal immunity have been shown to be lacking in C-section infants [126], and C-section has been associated with an increased risk for immune disorders, such as allergic rhinitis, asthma and celiac disease, among others [127,128]. Furthermore, in accordance with their more altered microbiota composition, infants born to females undergoing a repeat C-section without ruptured membranes have a risk of asthma that is increased by 60% over that of infants whose mothers presented with ruptured membranes and/or labor prior to the C-section [129]. Of note, in the case of mothers with a history of atopic disease, infants delivered by C-section are eight-fold more likely to develop allergies [130].

## 7. Effects of Breast Milk *vs.* Formula Feeding

Important differences have also been noted between the microbiotas of breast-fed and formula-fed infants, with breast-fed infants generally becoming colonized with higher numbers of bifidobacteria and lactobacilli [131]. In addition, increased species richness with overrepresentation of *Clostridium difficile* has been reported for formula-fed infants [124]. Breast milk likely plays several complementary roles in shaping the gut microbiota and the immune system of the infant. These include prebiotic actions, by which nutritional components of milk select for the growth of specific types of bacteria, as well as direct transfer of bacteria and immune components. The oligosaccharides of human milk (HMO), one of the main components of breast milk, are only partially digested in the small intestine and mostly reach the colon, where they are fermented, mainly by *Bifidobacterium*, to produce short-chain fatty acids leading to an acidification of the milieu. Therefore, HMOs have a clear prebiotic effect by stimulating selectively the development of a *Bifidobacterium*-rich microbiota [132]. In addition, breast milk contains numerous factors that modulate and promote the development of the infant immune system, including Igs, antimicrobial compounds, such as CD14, lysozyme and lactoferrin, immunoregulatory cytokines, such as TGF-β and IL-10, and lymphocytes expressing gut-homing markers [133,134]. Like the *in utero* environment, breast milk had been traditionally considered sterile in healthy women, but numerous studies have now demonstrated the presence of bacteria by both culture-dependent [12,135,136,137,138,139] and molecular techniques [137,138,139,140,141,142]. Breast milk from healthy women contains approximately 103–104 cfu/mL, and the number of cultivable bacterial species that have been isolated from a single woman ranges from two to 18 [143], although hundreds of species are detected by 16S rRNA pyrosequencing. Cultured genera include mostly lactic acid bacteria, such as *Lactobacillus*, *Leuconostoc*, *Streptococcus*, *Enterococcus*, *Lactococcus* and *Weissella*, as well as *Bifidobacterium* and the skin bacteria *Propionibacterium* and *Staphylococcus*. In addition, molecular methods indicate that Gram-negative bacteria are also present, including *Serratia*, *Pseudomonas* and some typical inhabitants of the oral cavity, such as *Veillonella*, *Leptotrichia*, and *Prevotella*. The overall composition of the milk microbiome changes along lactation, with oral bacteria increasing significantly from colostrum to mature milk, as well as with factors such as maternal weight and delivery mode [141].

In addition to their likely role as early gut colonizers, the live bacteria provided by breast milk, as well as their molecular components, probably serve to educate the infant’s immune system as to which bacteria should be considered habitual occurrences in the gut and which should be viewed as signs of danger [144]. Breast milk may facilitate the establishment of tolerance to the bacteria it carries through the concomitant transfer of immunosuppressive and antiinflammatory cytokines, such as IL-10 and TGF-β [145], as well as the promotion of IL-10 production in the infant [146]. Furthermore, the microbiota promoted by human breast milk also favors the development of the immune-protective function of the gut mucosa. The abundance of bifidobacteria, particularly *B. infantis*, during the first months of life has been directly associated with the levels of secretory IgA in the intestine [147] and *B. infantis* isolated from infants and cultured on HMOs has been shown to actively stimulate increased expression of tight-junction proteins and to provide anti-inflammatory effects [148]. Accordingly, numerous studies have demonstrated that breast-fed infants experience a lower incidence of infectious and inflammatory conditions, including NEC and neonatal sepsis, and have a lower risk of developing autoimmune diseases such as rheumatoid arthritis, celiac disease and type 1 diabetes [149]. On the other hand, the role of breast-feeding in preventing atopic disease remains controversial, as some studies demonstrate protective effects whereas others do not [134,150]. This might be due to heterogeneity in study design, but also to differences in the breast milk of different women, related to genetics, diet or health status, and to the length of breastfeeding. For instance, levels of IgA in breast milk vary, and an inverse association has been detected between them and the infant’s risk of developing atopic dermatitis at an early age [150]. Similarly, low levels of TGF-β in breast milk have been related to an increased risk of atopic illness in infants [151]. In addition, the family’s history of atopic disease and other risk factors condition the benefit of breastfeeding, which appears to be more pronounced in infants with atopic heredity [152].

Maternal diet is an important element affecting the composition of breast milk and can influence the levels of the immune factors that it carries, as well as the development of the infant’s immune cells. In particular, the fatty acids (FA) content of the maternal diet, reflected in breast milk, appears to carry special relevance for the development of immune function in the infant and his/her propensity to immune disease, especially in terms of tolerance to food antigens [149,153]. An important route through which breast milk FA exert their influence is by shaping the phospholipid composition of the infant’s cellular membranes, which, in the case of immune cells, strongly affects their function [153]. Additionally, TGF-β levels in breast milk have been reported to correlate positively with the content of polyunsaturated FA (PUFA) and negatively with that of saturated FA [151]. Furthermore, among the PUFA, the ratio of molecules of different chain lengths has been related to the infant’s risk of atopic illness [154]. For instance, cohort studies have demonstrated lower levels of n-3 PUFA in the milk of mothers whose infants would show symptoms of atopic illness before the age of 18 months [155].

## 8. Outlook: Possibilities for Prebiotic and Probiotic Supplementation

As we learn more about the processes that shape the microbial colonization of the gut and their effects on the development of the immune system, numerous avenues for potential modulation of the microbiota early in life are opening, with great promise for improving health. However, because of the complexity of the microbiota, the numerous routes through which it impacts on our biology and the many factors that affect its establishment, we are still far from reaching a knowledge base that would allow us to manipulate this community for precise therapeutic or preventive aims. Nevertheless, successes in this direction have started to take place. Of notice, late-pregnancy treatment of women having a family history of allergy with *Lactobacillus rhamnosus* LGG can lower the incidence of atopic disease in infants by 50% [156], and follow up of the infants has indicated that the protective effect is still apparent by seven years of age [157]. However, a later study has pointed out that the probiotic treatment is only effective in the atopy-prone infants who are born by C-section [158], which, as already mentioned, elevates enormously the risk for developing allergies [130].

The use of probiotics is proving to be particularly successful in preterm infants, where it is providing novel strategies to avoid the frequently devastating outcomes of immaturity and aberrant colonization of the gut, such as NEC. A large meta-analysis of probiotic therapy in premature infants with strains of *Bifidobacterium* or *Lactobacillus* [88], including 11 articles reporting clinical studies totaling 2176 participating infants, showed that the risk for NEC and death was lowered by 30% by probiotic use, although sepsis did not differ from the control groups. Importantly, no significant adverse effects were reported in this analysis, and other studies that have followed very low birth weight (<1500 g) neonates have also shown long-term safety for up to two years.

Recent studies are also supporting beneficial effects for prebiotic supplementation of infant formulas aimed at promoting a *Bifidobacterium*-rich ‘breast-fed-like’ microbiota. Although the complex mixture of breast milk HMOs cannot be artificially reproduced in infant formulas [159], utilization of prebiotic compounds, such as inulin, plant-derived fructooligosaccharides (FOS) or enzymatically-synthesized galactooligosaccharides (GOS), may emulate some of the benefits of breast milk. Randomized controlled trials (RCT) have demonstrated bifidogenic effects in infants both for FOS and for mixtures of GOS with FOS or inulin [160], and a systematic review of RCTs has shown that prebiotic-supplemented formula is generally well tolerated [161]. Importantly, early supplementation of formula with a mixture of short chain GOS and long chain FOS had prebiotic and immunomodulatory effects comparable to those of HMOs and reduced the incidence of allergic manifestations and infections during the first two years of life [162,163]. In contrast, a recent RCT review of 12 studies revealed no impact of prebiotic supplementation of formula on the abundance of bifidobacteria and lactobacilli in infant stools [164]. However, it is necessary to consider that the studies included in this review employed a variety of prebiotics, doses and administration regimes.

Furthermore, it is likely that the prebiotic compounds currently used in formula are not the best suited to emulate the functions of HMOs. HMOs are complex glycans composed of five different monosaccharides, whereas FOS and GOS are much simpler structures [165]. Possibly as a result of these differences, the effects of FOS and GOS on the growth of *Bifidobacterium* are not identical to those of HMOs. Firstly, growth on HMOs is restricted to select bifidobacteria, primarily *B. infantis* and *B. bifidum*, which possess the specific fucosidase and sialidase functions required to deconstruct the HMO polymer, whereas FOS and GOS enable the growth of a wider array of bifidobacteria [159]. Moreover, in gene expression analyses of *B. infantis* grown with FOS and GOS, a very different set of genes was activated as compared to growth on HMOs [166]. Therefore, it may be worthwhile to explore a larger selection of the prebiotic compounds that are already commercially available [167] for potential use in infant formulas. Another promising venue is the investigation of the prebiotic capacities of oligosaccharides present in the milk of different animals [159]. Bovine and goat milk oligosaccharides (BMOs and GMOs) are likely prebiotic candidates, as, although they also are less complex, they seem to share sufficient structural properties with HMOs to be able to carry out similar functions. Goat milk is especially rich in complex lactose-derived oligosaccharides and contains fucosylated species [168], which are common in HMOs [169] but not in BMOs [170]. Importantly, it has been demonstrated that administration of GMOs in a rat model of colitis and IBD ameliorates the gut microbiota and the intestinal function, while reducing inflammation, colonic lesions and body weight loss [171]. Ongoing advances in glycomics will shed light on the specific structural elements of HMOs that elicit their biological functions, thus, further informing the choice of oligosaccharides from other sources for prebiotic use [159].

## 9. Concluding Remarks

The alteration of gut microbiota development patterns during infancy can have a variety of negative effects on immune health, which are likely to endure for long periods of time. Moreover, factors that may produce such alteration are often encountered in modern life, including early exposure to antibiotics, birth by C-section and lack or insufficient duration of breast-feeding. In order to devise strategies that may paliate the effects of such factors, it is imperative to further our understanding of the succession of events that result in the establishment of a well-balanced gut microbiota in the infant, and of the complex interactions with the host immune system that are laid out during this process. Although our knowledge of these issues is far from complete, the current state-of-the-art indicates that the influence of the gut microbiota on immune development and health may start *in utero*, so that strategies aimed at the favorable priming of the immune system may need to start during pregnancy. The use of prebiotics or probiotics for supplementation of infant formulas and for preventive or restorative therapies in cases of early antibiotic use also holds great promise. However, the optimal display of such approaches will necessitate much further research into the joint development of the gut microbiota and the immune system during infancy.
